# From “traction bronchiectasis” to honeycombing in idiopathic pulmonary fibrosis: A spectrum of bronchiolar remodeling also in radiology?

**DOI:** 10.1186/s12890-016-0245-x

**Published:** 2016-05-23

**Authors:** Sara Piciucchi, Sara Tomassetti, Claudia Ravaglia, Christian Gurioli, Carlo Gurioli, Alessandra Dubini, Angelo Carloni, Marco Chilosi, Thomas V Colby, Venerino Poletti

**Affiliations:** Department of Radiology, Azienda USL Romagna, Ospedale GB Morgagni, Via C. Forlanini, Forlì, FC 34-47121 Italy; Department of Diseases of the Thorax, Azienda USL Romagna, Ospedale GB Morgagni, Forlì, Italy; Department of Pathology, Azienda USL Romagna, Ospedale GB Morgagni, Forlì, Italy; Department of Radiology, Ospedale Santa Maria, Terni, Italy; Department of Pathology and Diagnostics, University of Verona, Verona, Italy; Department of Laboratory Medicine and Pathology, Mayo Clinic, Scottsdale, AZ USA; Department of Respiratory Diseases & Allergy, Aarhus University Hospital, Aarhus, Denmark

**Keywords:** Traction bronchiectasis, Honeycombing, Fibroblastic Foci, Bronchiolar dysplastic proliferation

## Abstract

**Background:**

The diagnostic and prognostic impact of traction bronchiectasis on high resolution CT scan (HRCT) in patients suspected to have idiopathic pulmonary fibrosis (IPF) is increasing significantly.

**Main body:**

Recent data demonstrated that cysts in honeycombing areas are covered by epithelium expressing bronchiolar markers. In IPF bronchiolization is the final consequence of a variety of pathogenic events starting from alveolar stem cell exhaustion, and ending in a abnormal/dysplastic proliferation of bronchiolar epithelium. CT scan features of traction bronchiectasis and honeycombing should be interpreted under the light of these new pathogenetic and morphologic considerations.

**Short conclusion:**

We suggest that in IPF subjects traction bronchiectasis and honeycombing -now defined as distinct entities on HRCT scan- are actually diverse aspects of a continuous spectrum of lung remodeling.

## Background

Histologically, Usual Interstitial Pneumonia (UIP) is characterized by a combination of “patchy fibrosis” and fibroblastic foci displaying a “patchwork pattern”. Disease progression is characterized by the appearance of airspaces lined by plump cuboidal or even ciliated columnar cells showing a immunohistochemical and molecular bronchiolar phenotype [1–3].

In the recent years, the role of bronchiolar epithelium in the development of UIP pattern (in IPF subjects) has been widely emphasized [4–8].

Factors leading to lung remodeling include senescence, alveolar stem cell exhaustion and consequently aberrant activation of the wnt-β-catenin and hedgehog pathways that normally regulate branching morphogenesis in the lungs [6, 9].

Mechanical stress may contribute to the subpleural and usually basilar localization of UIP changes [10, 11].

The final stage of this “bronchiolization” process corresponds radiologically to honeycombing, typically seen first in the subpleural regions of the lower lobes [2, 3].

## Main text

By CT, a “definite” usual interstitial pneumonia (UIP) pattern, as seen in IPF, is characterized by the presence of reticulation, traction bronchiectasis and honeycombing in a basal and peripheral predominant distribution. The presence of honeycombing and traction bronchiectasis, besides reticulation, is crucial [12–19].

However if we reconsider morphological aspects in light of the pathogenic events discussed above, we identify some interesting key points in the interpretation of CT findings.

First, the sites in which mechanical stress and remodeling are highest, correspond to the areas in which traction bronchiectasis and honeycombing appear.

**Fig. 1:**
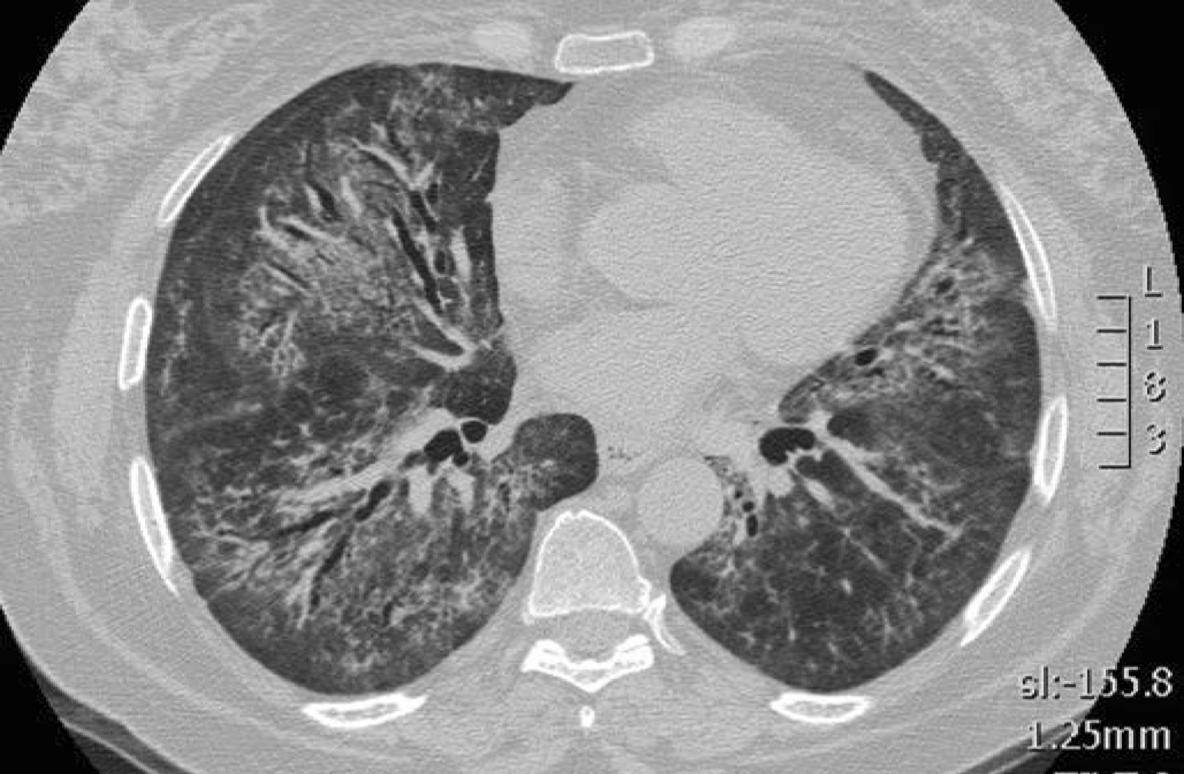
CT scan of a 52 years old lady, affected by idiopathic NSIP. Bilateral, peribronchovascular ground glass attenuation, due to intralobular fibrotic changes. Traction bronchiectasis are present bilaterally surrounded by ground glass,“fibrotic” attenuation, mainly in the right middle lobe and in both lower lobes. No honeycombing is present. A relative subpleural sparing is also visible

Second, most of the “scarred” tissue is in the region distal to the traction bronchiectasis, beneath the pleura, and does not concentrically surround the dilated bronchi. Conversely in nonspecific Interstitial Pneumonia (NSIP), traction bronchiectasis is completely surrounded by the fibrotic tissue (Fig. 1).

**Fig. 2:**
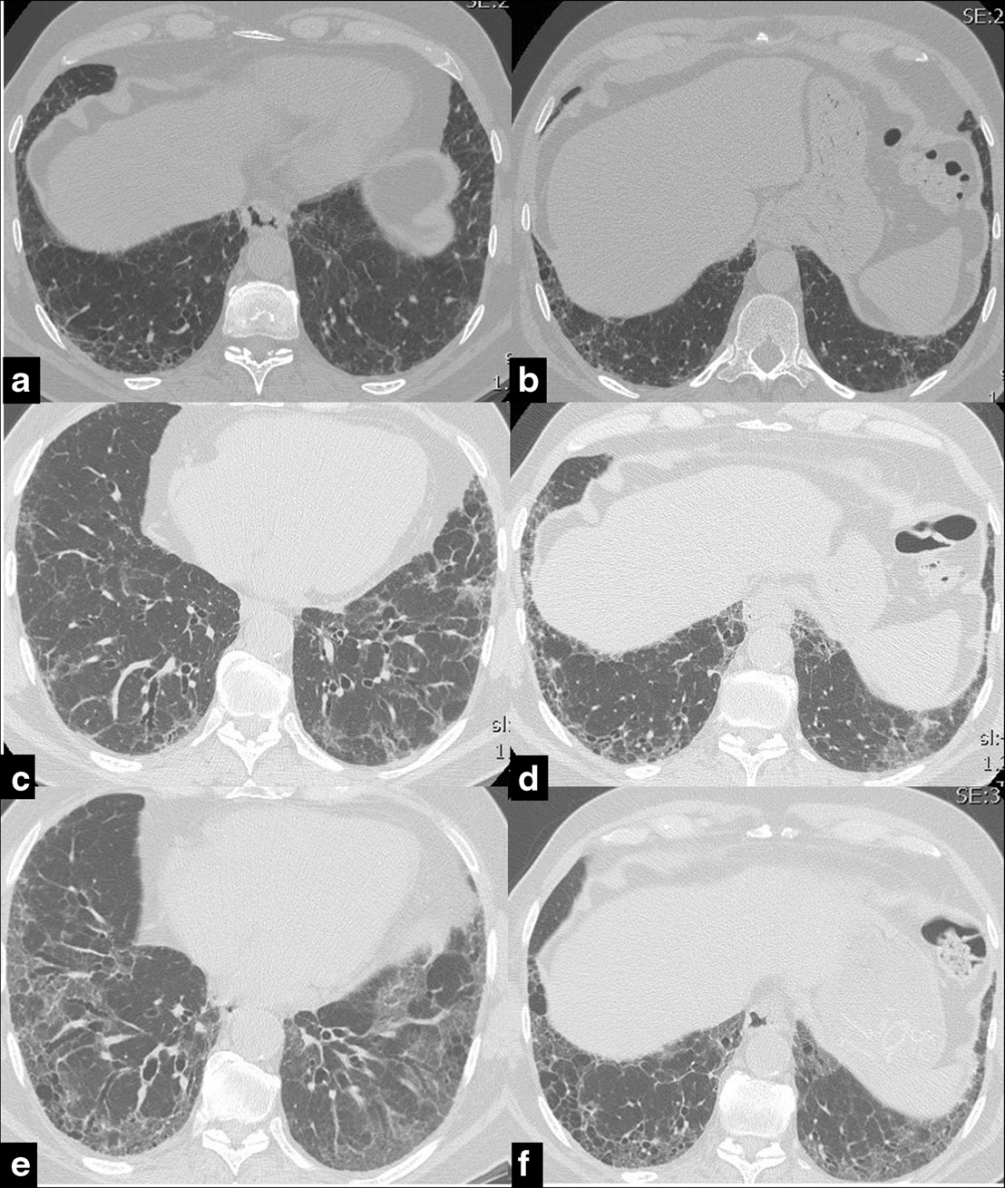
Serial CT images of a 63 years old male affected by IPF. UIP pattern has been diagnosed through surgical lung biopsy at diagnosis. CT shows the progressive worsening of the coarseness. In 2007 (**a**, **b**) a moderate peripheral fibrotic reticulation is present. In the following years it progressively gets worse, particularly in the right lower lobe, with increase of the extension of traction bronchiectasis in 2010 (**c**, **d**) and with honeycombing and traction bronchiectasis in 2014 (**e**, **f**)

Furthermore as the fibrosis progresses, dilatation of the airways increases in severity from the periphery to the inner third of the lungs, passing from a score 1 (more peripheral) to 3 (from periphery to the inner third of the parenchyma) [score proposed by Edey et al. [20]] (Fig.2).

In CT scans, when fibrosis is more severe, bronchiectasis tend to follow the convexity of the pleura and to overlap with honeycombing features [21].

Therefore, also in CT scans, traction bronchiectasis observed in IPF subjects is better interpreted as resulting from bronchiolar proliferation rather than from pure mechanical traction of a single airway by scarring tissue. Supporting this point of view is the recent observation by Staats et al. [4] on explanted lungs obtained from patients with IPF. They showed a positive correlation between honeycombing assessed by CT and bronchiolectasis (p = 0.001) and respiratory-lined cysts (p = 0.001) histologically counted. If we consider traction bronchiectasis as the solely consequence of pure mechanical traction around the airway, this process should result in a relatively stable number of traction “holes” with an enlargement of lumen reaching the periphery, as actually is present only in NSIP. Thus, in IPF, remodeling process appears to be a continuum from traction bronchiectasis to honeycombing and conceptual separation of the two processes may be misleading.

Walsh et Al. [19] reinforced this concept recently. The authors retrospectively reviewed radiological features of 162 biopsy proven cases of UIP and NSIP, delineating a radiological visual score for each case and correlating these radiological data to the fibroblastic foci profusion score and other morphologic aspects. They concluded that in UIP there was a strong correlation between traction bronchiectasis, honeycomb changes and fibroblastic foci profusion. In conclusion, according to the “alveolar stem cell exhaustion” model explaining at least part of the pathogenic events in IPF [14] we suggest that traction bronchiectasis and honeycombing is a unique and continuous process of bronchiolar dysplastic proliferation and to interpret accordingly the HRCT features.

## Conclusions

In agreement to the “alveolar stem cell exhaustion pathogenic model” in IPF, we may begin to consider traction bronchiectasis and honeycombing as a unique and continuous process of bronchiolar proliferation also in HRCT scan.

## Abbreviations

UIP: Usual Interstitial Pneumonia
NSIP: non specific interstitial pneumonia
IPF: idiopathic pulmonary fibrosis
HRCT: high resolution computed tomography
